# Characterization of Visual Function, Interocular Variability and Progression Using Static Perimetry–Derived Metrics in *RPGR*-Associated Retinopathy

**DOI:** 10.1167/iovs.17-23739

**Published:** 2018-05

**Authors:** James J. L. Tee, Yesa Yang, Angelos Kalitzeos, Andrew Webster, James Bainbridge, Richard G. Weleber, Michel Michaelides

**Affiliations:** 1 UCL Institute of Ophthalmology, University College London, London, United Kingdom; 2 Moorfields Eye Hospital, London, United Kingdom; 3 Casey Eye Institute, Oregon Health & Science University, Portland, Oregon, United States

**Keywords:** retinitis pigmentosa, genetic diseases, static perimetry, visual field, RPGR

## Abstract

**Purpose:**

To characterize bilateral visual function, interocular variability and progression by using static perimetry–derived volumetric and pointwise metrics in subjects with retinitis pigmentosa associated with mutations in the retinitis pigmentosa GTPase regulator (*RPGR*) gene.

**Methods:**

This was a prospective longitudinal observational study of 47 genetically confirmed subjects. Visual function was assessed with ETDRS and Pelli-Robson charts; and Octopus 900 static perimetry using a customized, radially oriented 185-point grid. Three-dimensional hill-of-vision topographic models were produced and interrogated with the Visual Field Modeling and Analysis software to obtain three volumetric metrics: V_Total,_ V_30_, and V_5_. These were analyzed together with Octopus mean sensitivity values. Interocular differences were assessed with the Bland-Altman method. Metric-specific exponential decline rates were calculated.

**Results:**

Baseline symmetry was demonstrated by relative interocular difference values of 1% for V_Total_ and 8% with V_30_. Degree of symmetry varied between subjects and was quantified with the subject percentage interocular difference (SPID). SPID was 16% for V_Total_ and 17% for V_30_. Interocular symmetry in progression was greatest when quantified by V_Total_ and V_30,_ with 73% and 64% of subjects possessing interocular rate differences smaller in magnitude than respective annual progression rates. Functional decline was evident with increasing age. An overall annual exponential decline of 6% was evident with both V_Total_ and V_30_.

**Conclusions:**

In general, good interocular symmetry exists; however, there was both variation between subjects and with the use of various metrics. Our findings will guide patient selection and design of *RPGR* treatment trials, and provide clinicians with specific prognostic information to offer patients affected by this condition.

Retinitis pigmentosa (RP) is an entity that describes a group of genetically heterogeneous disorders typically presenting with nyctalopia, followed by peripheral vision loss that extends concentrically to affect central vision at later stages of disease.^1,2^ RP is estimated to affect up to 1:3000 individuals worldwide^1–4^ and accounts for a large part of the inherited retinal disorders that are now the commonest cause of visual impairment in working-age adults and second commonest cause in childhood.^5^

Thirty to 40% of RP cases can be attributed to an autosomal dominant mode of inheritance; 45% to 60%, to an autosomal recessive mode; and 5% to 15%, to an X-linked (XL) mode.^1,6^ In around 75% of cases, XLRP is caused by pathogenic sequence variants within the retinitis pigmentosa GTPase regulator gene (*RPGR*). *RPGR*-associated RP is among the most severe forms of RP owing to its early childhood onset and severity of progression,^7^ and is currently an important target of gene therapy trials following recent successes in animal models^8,9^ (NCT03116113 at Oxford Eye Hospital, Oxford, UK; and NCT03252847 at Moorfields Eye Hospital, London, UK).

Perimetry is widely used as a means of evaluating visual function in RP. Kinetic Goldmann perimetry has been used for this purpose.^10–20^ Static perimetry has been used less frequently; however, its use is gaining ground over kinetic perimetry both in observational studies and treatment trials of RP.^21–26^ We have recently pioneered the use of a custom-made test grid for RP, using Octopus 900 static perimetry, together with the use of Visual Field Modeling and Analysis (VFMA) software to interrogate test results for further volumetric analyses.^27,28^

Contrast sensitivity (CS) has been demonstrated to be a useful and valid measure of visual function in patients with RP.^29–34^ Reduction in CS levels are associated with difficulties in daily activities—in particular tasks requiring distance judgment, driving, and mobility—as compared to visual acuity (VA), which is associated with tasks requiring good visual resolution and adaptation to varying light levels.^35^ VA being a standard and widely used functional metric is also a favored primary outcome of many treatment studies.^36–38^ CS, VA, and visual field extent have all been shown to correlate strongly with mobility in patients with RP.^33,34^ Close to 70% of variance in mobility performance is associated with visual function as characterized by these three modalities.^33,34^ The association between CS and disability is thought to be independent of VA despite correlations between CS and VA performance.^35,39^ In patients with RP, functional abnormalities can be detected on CS testing in the presence of normal VA levels.^29–31^

The lack of high-quality prospective protocol-driven functional data to elucidate progression as part of the natural histories of the various genetic disorders within the RP family is a limiting factor in providing accurate prognostic information to patients in clinic, and furthermore hinders efforts to determine the suitability of patients for gene therapy trials as well as methods of assessing treatment outcomes. With the exception of a few retrospective studies on *RPGR*-associated retinopathy,^17–20^ previous studies either have included subjects with different forms of inheritance that were collectively studied and analyzed,^10–12,14,15,23^ or lack molecular confirmation of disease-causing genes despite efforts to separately describe subjects by inheritance patterns,^13,16,21^ thus rendering these studies inherently limited for the aforementioned prognostic and therapeutic needs.

As such, we set out to investigate visual function in this prospective study comprised solely of RP subjects with molecularly confirmed pathogenic *RPGR* sequence variants, with the following aims: (1) to characterize visual function at baseline with static perimetry (volumetric and pointwise metrics), VA, and CS metrics; (2) to characterize progression rates with similar metrics; (3) to ascertain the degree of interocular symmetry of function at baseline; (4) to ascertain the degree of interocular symmetry with respect to progression; (5) to establish indices to quantify symmetry to guide upcoming treatment trials; (6) to investigate the effects of age and genotype on baseline function; (7) to describe correlations between baseline function, progression rates, and age; and (8) to determine overall exponential rates of progression with each metric.

## Patients and Methods

### Patients

**Figure 1:**
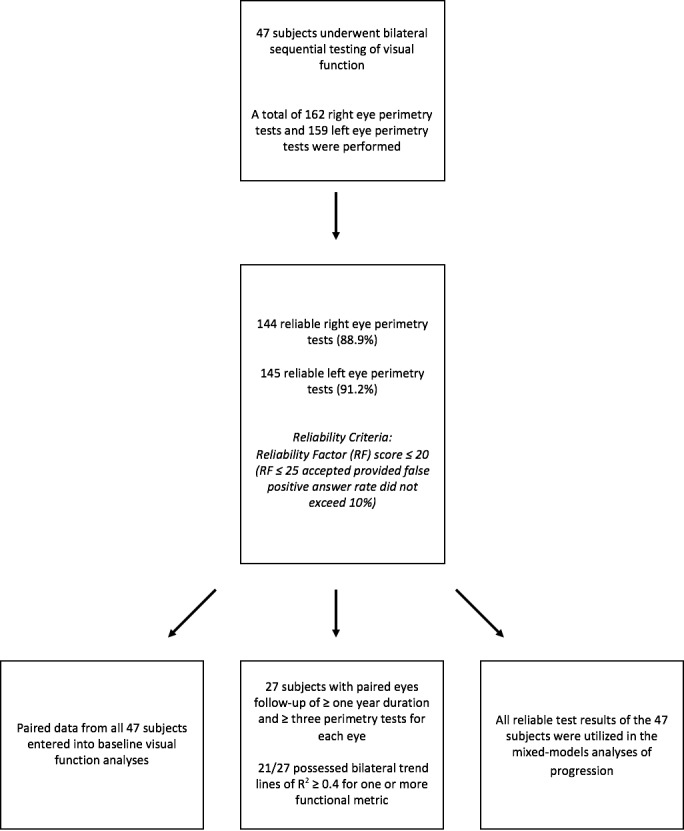
Flowchart illustrating recruitment of subjects and study data based on the inclusion criteria.

Ethical approval was received from the ethics committee at Moorfields Eye Hospital for this prospective observational study. The Declaration of Helsinki was adhered to throughout the study. All 47 subjects were affected males with RP, with genetic confirmation of disease-causing variants in *RPGR*. The criteria for inclusion are detailed in Figure 1.

### Methods

At each visit, assessments of visual function commenced with best-corrected visual acuity (BCVA) at 4 m, using the Early Treatment Diabetic Retinopathy Study chart followed by CS testing at a distance of 1 m with the Pelli-Robson chart. BCVA was recorded in logarithm of the minimum angle of resolution (logMAR) units and CS as logCS units.

Automated white-on-white static perimetry testing with the Octopus 900 (Haag-Streit AG, Köniz, Switzerland) was performed sequentially on right eyes followed by left eyes of subjects, with fixation monitored closely throughout the test by a dedicated ophthalmic technician in the clinical research facility. Background illumination was set at 10 cd/m^2^ (31.4 apostilbs [abs]). Full threshold testing was performed with the GATE strategy^40,41^ using Goldmann size V stimulus of 4000-abs brightness and 200-ms duration. A customized, radially oriented 185-point grid with central condensation of test points and good peripheral coverage extending radially 55.5° nasally and superiorly, 67° inferiorly, and 80° temporally was used for all tests.

Only data from reliable tests were used for analysis. Test reliability was determined by the reliability factor (RF), which is the sum of false-positive and false-negative answers divided by the total number of positive and negative catch trials presented. The RF was expressed as a percentage score. Tests were deemed reliable if the RF was ≤20. Tests with RF scores between 21 and 25 were included provided the false-positive answer rate for these tests did not exceed 10%. Tests with RF scores greater than 25 were automatically excluded from analysis (Fig. 1).

Octopus mean sensitivity (OMS) values for each test were obtained by using Octopus Eyesuite vendor software. Following this, test data were exported from the Eyesuite platform as comma-separated value (csv) files for further analysis with VFMA software. This program generates a three-dimensional representation of the hill-of-vision of the visual field with the solid angle (in unit steradian) of the base of the hill defined by the outer perimeter of the peripheral test locations in the test grid and the sensitivity as the *y*-axis, thereby allowing calculation of the volume of sensitivity beneath the surface in unit decibel-steradian (dB-sr). In comparison to the mean sensitivity metric, which provides an average value of retinal sensitivity, volumetric metrics characterize the total amount of sensitivity in the entire hill-of-vision, V_Total_, or any subregion of interest as defined by a geometric selection, such as a circular selection or a selection defined by topographic isosensitivity lines or a subretinal injection site. The three volumetric metrics used in this study were V_Total_ (volumetric analysis of total visual field captured by the entire grid); V_30_ (analysis of the visual field contained within a central circle of 30° radius); and V_5_ (analysis of the visual field contained within a central circle of 5° radius).

### Statistical Analysis

**Figure 2:**
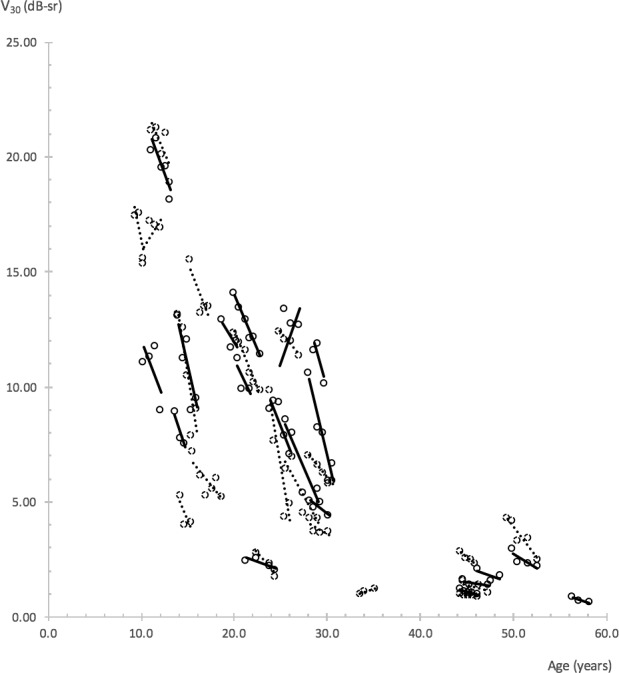
Linear trend lines illustrating V_30_ progression rates. Trend lines and data points of right eyes are represented by solid slope lines and solid circles, left eyes by dotted slope lines and dotted circles. Trend lines with R^2^ ≥ 0.4 are shown. Perimetry tests that met reliability criteria were used. A minimum of three tests with follow-up duration ≥1 year were required of each eye.

Progression rates for individual eyes were obtained from the gradients of linear trend lines fitted to data points, using a least squares method (Microsoft Excel for Mac, Version 15.24; Microsoft, Redmond, WA, USA). Data were plotted separately for each metric against subject age at time of testing. Individual trend lines were fitted for each eye of each subject who had undergone a minimum of three perimetry visits over a minimum period of 1 year (an example is shown in Fig. 2). The *R*^2^ value for each trend line was inspected and only individual rates with *R*^2^ ≥ 0.4 were included for further analyses.

Statistical analysis was carried out with XLSTAT version 18.07 software (Addinsoft, New York, NY, USA). Data on visual function at baseline and rates of progression are presented for all eyes, as well as by laterality (Tables 1, 2). Data are expressed as mean values ± standard deviation (SD). Median and interquartile range (difference between first and third quartiles) are also provided for nonnormally distributed data.

**Table 1:**
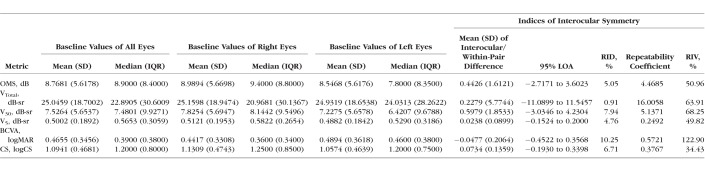
Baseline Metric Values and Indices of Interocular Symmetry of All Subjects

**Table 2:**
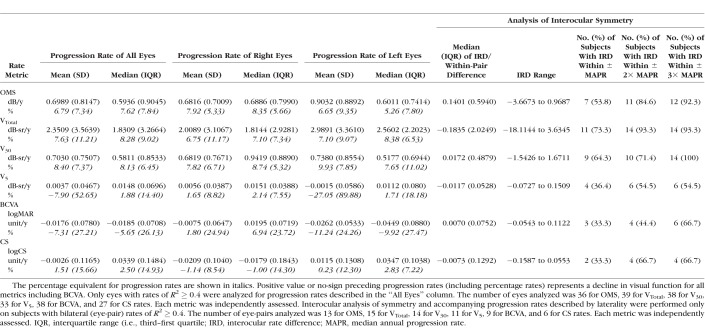
Progression Rates With Analysis of Interocular Rate Symmetry for All Study Metrics

The Bland-Altman method was used to assess interocular differences in baseline function and progression rates as characterized by each study metric. As the interocular differences for baseline function were normally distributed, the mean ± SD of interocular differences (equivalent of Bland-Altman within-pair differences) and 95% limits of agreement (LOA) were calculated. Interocular differences were obtained by right eye minus left eye values (Table 1). For each metric, the mean of interocular differences expressed as a fraction of the mean baseline value of all eyes was calculated and presented as the relative interocular difference (RID). A second index of interocular symmetry, the relative interocular variability (RIV), was calculated by expressing the repeatability coefficient (repeatability coefficient = 1.96 × √2 × SD) as a fraction of the mean baseline value of all eyes. The RID and RIV were calculated to facilitate further comparisons between the various metrics. A third index of symmetry, termed the “subject percentage interocular difference” (SPID), was calculated for each metric to further quantify the degree of interocular differences at baseline that existed within subjects in our cohort (Table 3). The SPID was obtained by expressing each subject's baseline interocular difference as a fraction of his or her own baseline value, which in turn was calculated as the average of baseline values for both eyes of the subject. The actual magnitude of difference was obtained as the SPID irrespective of positive or negative values. The RID, RIV, and SPID are presented in percentage form by multiplication with 100.

**Table 3:**
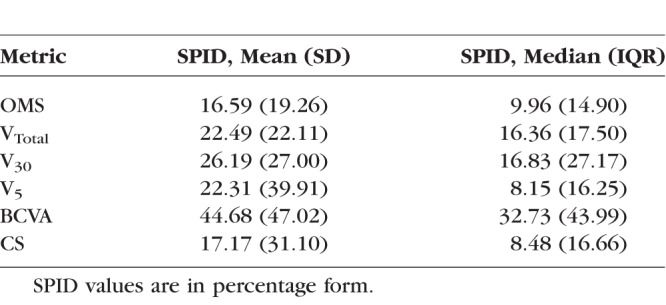
SPID Values Calculated at Baseline for Each Study Metric

Interocular analyses of symmetry for progression rates with each metric were performed by using data from subjects who possessed bilateral trend lines of *R*^2^ ≥ 0.4. Owing to the nonparametric distribution and smaller number of interocular progression rates, a nonparametric approach with appropriate descriptive statistical terms was used for the Bland-Altman analysis (Table 2). The median, range, and interquartile range of interocular (within-pair) differences were calculated, together with the proportion of subjects with interocular rate differences that fell within specific reference values.^42^ These reference values were obtained from our annual progression rates.

Spearman's correlation coefficient was used to investigate the following: (1) interocular correlation in baseline function; (2) interocular correlation in progression rates; (3) correlation between age and baseline function of all eyes; (4) correlation between BCVA and other metrics at baseline for all eyes; (5) correlation between CS and other metrics at baseline for all eyes; and correlation between SPID and visual function for all metrics at baseline (visual function taken as the average value of right and left eyes as characterized by each metric); (6) correlation between SPID and age at baseline; (7) correlation between progression rate and baseline visual function for all metrics; and (8) correlation between progression rate and age at baseline for all metrics. Results are shown in Table 4.

**Table 4:**
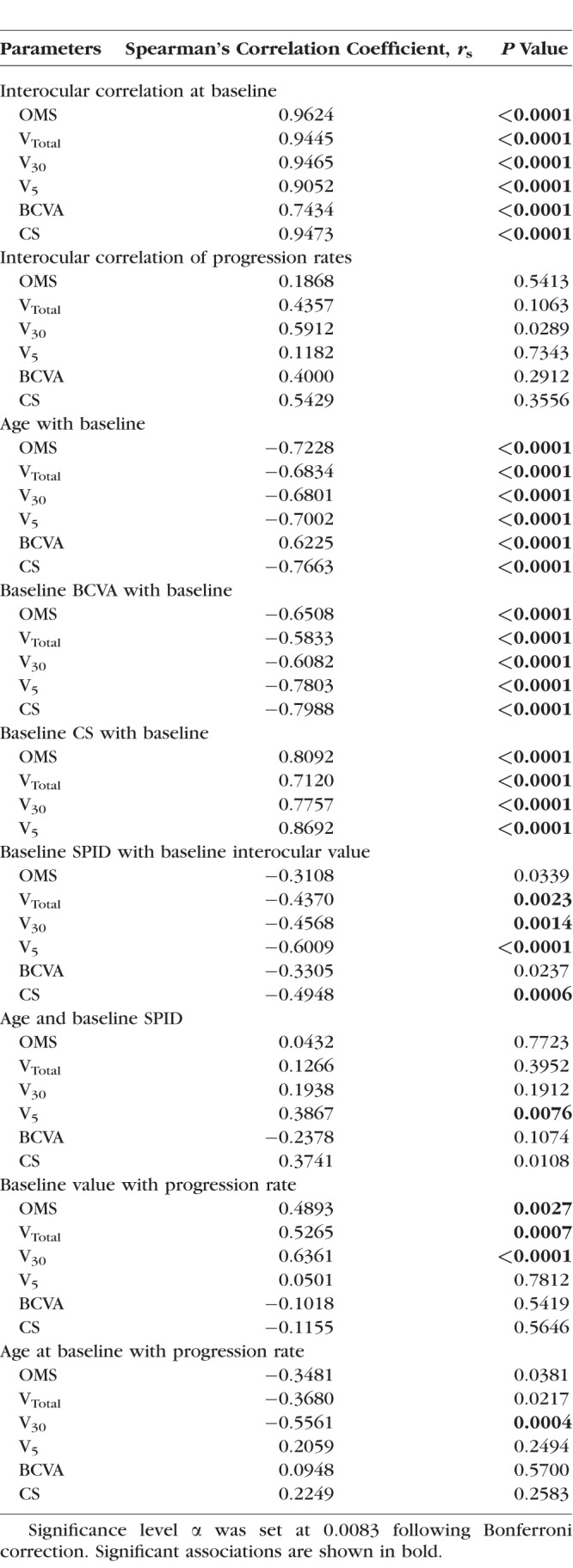
Investigated Associations of Interocular Correlation, Baseline Function, Progression, and Age With Study Metrics

The effects of age, genotype, and age-genotype interaction on baseline visual function were investigated with a 2-way ANOVA (Table 5). Subject age was calculated from birth to time at baseline visit. Age was divided into five categories: category 1: <15 years, category 2: 15 to <20 years, category 3: 20 to <25 years, category 4: 25 to <30 years, category 5: ≥30 years of age. Genotype was divided into two categories depending on the position of the *RPGR* sequence variant: Exon 1-14 or open reading frame 15 (ORF15) variants. The distributions of ANOVA residuals were inspected for normality. Post hoc multiple pairwise comparisons were performed with Tukey's test in instances of a significant ANOVA result.

**Table 5:**
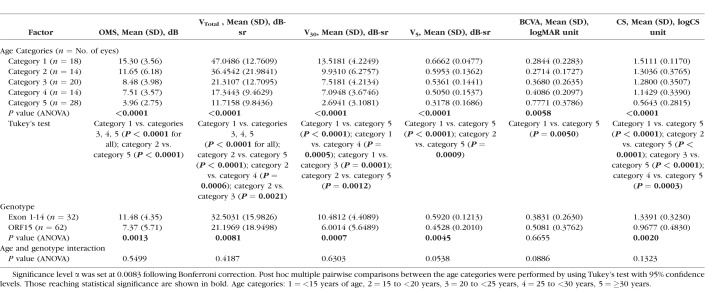
Results of a 2-Way ANOVA Investigating the Effects of Age and Genotype on Visual Function at Baseline as Characterized by Study Metrics

A mixed-models method was used to deduce overall rates of progression for each of the six metrics (Table 6). Analysis was performed with subjects' age (from birth to time of visual function testing) designated as a fixed effects quantitative explanatory variable. Each eye of each subject was selected as a random effects variable. Each metric was analyzed in turn as the dependent variable, with the analysis performed three times for each metric: “overall” refers to analysis performed before categorization of eyes into respective genotype groups; Exon 1-14 and ORF15 analyses were performed after categorization into the two genotype groups. The distributions of model residuals were inspected for normality.

**Table 6:**
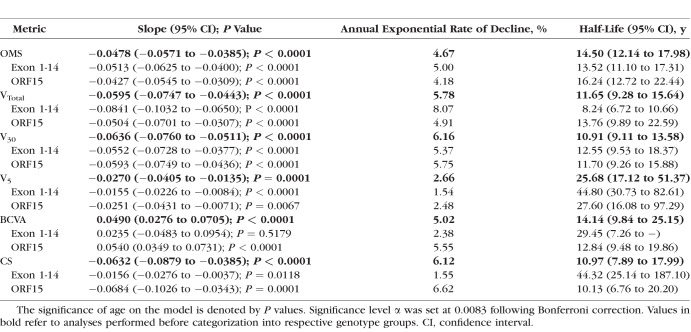
Indices of Overall Progression Represented by the Annual Exponential Decline Rate and Half-Lives, Calculated From Slope Values Obtained by a Mixed-Models Method for Each Metric With Age Designated as a Fixed Effects Variable

Photoreceptor degeneration in animal models has been shown to occur in an exponential fashion.^43^ Functional decline in RP patients over the long term has been well characterized with an exponential model.^13–17,44^ In addition, studies that have used static perimetry data to model progression in glaucoma have demonstrated that an exponential model of decay performs well at predicting future loss of function and provides a better fit, particularly for longer periods of follow-up.^45–47^ Thus, all values were converted into natural log form before analyses with the mixed-models method in order to model an exponential decline. Slopes indicating progression rates were obtained from computed model parameters with corresponding *P* values given to demonstrate the significance of age as an effect on the models. Half-lives with 95% confidence intervals were calculated by using the equation *t*_1/2_ = − log_e_ (2)/k for CS and perimetry-derived metrics; or in the case of BCVA, *t*_1/2_ = log_e_ (2)/k. Significance level α for all statistical tests was set at 0.0083 following Bonferroni correction for multiple simultaneous analyses on six metrics.

## Results

Six metrics were investigated at baseline and over time: (1) BCVA and CS; and (2) four static perimetry–derived retinal sensitivity metrics: OMS, V_Total_, V_30_, and V_5_. Reliability parameters of perimetry tests are provided in Table 7.

**Table 7:**
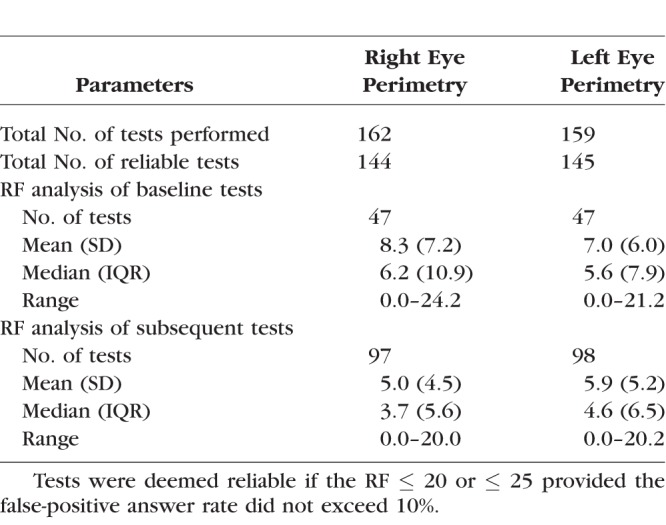
Reliability Parameters for Perimetry Tests

### Cohort Characteristics for Baseline Analysis

Forty-seven subjects (Fig. 1) underwent bilateral visual function assessments with BCVA, CS, and perimetry. Mean (SD) age of subjects at baseline was 25.83 (12.12) years (median [interquartile range, IQR]: 24.70 [17.73], range: 8.98–56.27 years). Sixteen subjects harbored mutations in Exon 1-14 and the remaining 31 subjects had variants in ORF15. Baseline values are provided in Table 1 with eyes grouped together, and separated by laterality to aid further analyses of interocular symmetry.

### Cohort Characteristics for Progression Rates Analyses

Thirty-seven subjects had BCVA and CS measurements performed over a period ≥ 1-year duration with measurements collected over ≥3 time points. Mean (SD) follow-up period for the 37 subjects was 2.12 (0.66) years (range: 1.01–3.51 years). Nine of 37 (24.3%) subjects possessed bilateral trend lines with *R*^2^ ≥ 0.4 for purposes of interocular BCVA comparisons. Six of 37 (16.2%) subjects possessed bilateral trend lines with *R*^2^ ≥ 0.4 for interocular CS comparisons.

Twenty-seven subjects had bilateral reliable visual field tests performed over ≥3 time points, with follow-up spanning a period ≥ 1-year duration. Mean (SD) follow-up period for the 27 subjects was 2.09 (0.69) years (range: 1.01–3.51 years). Twenty-one of 27 subjects possessed bilateral trend lines with *R*^2^ ≥ 0.4 for one or more perimetry-derived metrics for purposes of interocular progression rates analysis (Fig. 1). Fifteen subjects (55.6 %) possessed bilateral trend lines with *R*^2^ ≥ 0.4 for V_Total_; 14 subjects (51.9 %), with *R*^2^ ≥ 0.4 for V_30_; 13 subjects (48.1 %), with *R*^2^ ≥ 0.4 for OMS; and 11 subjects (40.7 %), with *R*^2^ ≥ 0.4 for V_5_ (Table 2).

### Baseline Values and Investigations of Interocular Symmetry

**Figure 3:**
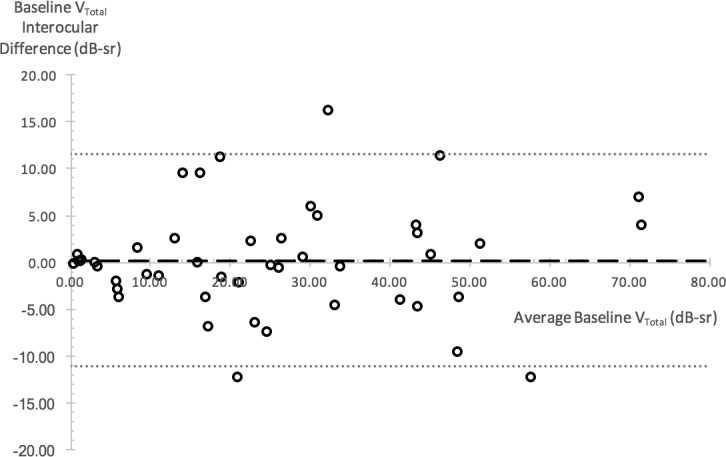
Bland-Altman plot illustrating V_Total_ interocular differences at baseline. Interocular difference is plotted against the average interocular value for each subject as represented by a circular data point. Dashed line shows mean of interocular differences; dotted lines represent upper and lower 95% limits of agreement.

Table 1 describes baseline values for all six metrics. Interocular correlation of baseline values for all metrics was strong as evident by Spearman's correlation coefficient *r*_s_ ≥ 0.9 for all metrics, except for BCVA (*r*_s_ = 0.74). All correlations were statistically significant at *P* < 0.0001 (Table 4). Interocular symmetry at baseline was investigated with the Bland-Altman method (Fig. 3 as example) with results provided in Table 1. In general, the mean interocular differences were small, demonstrating a high degree of symmetry between eyes, especially for perimetry-derived metrics. RID was smallest (1%) when visual function was assessed with V_Total_, increasing to 5% with OMS and V_5_, 7% with CS, 8% with V_30_, and to a maximum RID of 10% with BCVA. RIV was least with CS (34%) and greatest with BCVA (123%). RIV for V_5_, OMS, V_Total_, and V_30_ metrics was in between at 50%, 51%, 64%, and 68%, respectively.

Given the finding of significant variability in interocular differences among our subjects, we proceeded to calculate the SPID to quantify the degree of interocular differences at baseline with each metric (Table 3). Median SPID varied from 8.15%, 8.48%, and 9.96% for V_5_, CS, and OMS, respectively, to 16.36% for V_Total_ and 16.83% for V_30_. Median SPID was greatest with BCVA at 32.73%.

### Progression Rates and Investigations of Interocular Symmetry

Table 2 describes progression rates obtained with all six metrics. Greatest median rates of annual decline were obtained with V_Total_ and V_30_ metrics, at 8.28% and 8.13%, respectively; followed by rates obtained with OMS (7.62%), CS (2.50%), and V_5_ (1.88%). Median BCVA rate, however, showed a 5.65% annual improvement, which was equivalent to an annual improvement of 0.02 logMAR units.

A moderate interocular correlation was present for V_30_ rates (*r*_s_ = 0.59, *P* < 0.0289); however, this was no longer significant after Bonferroni correction (Table 4). In general, interocular correlation of progression rates was not significant, in contrast to the interocular correlation evident for baseline function.

**Figure 4:**
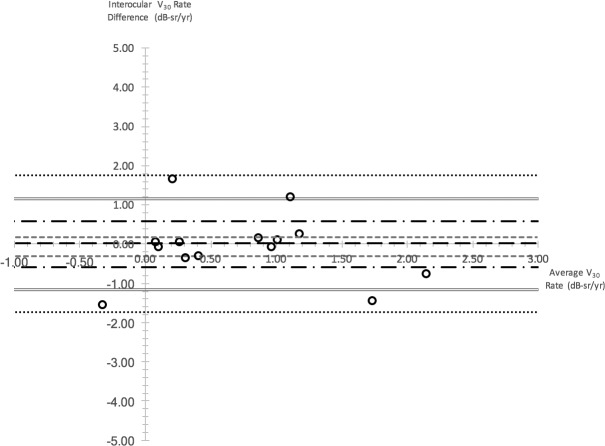
Bland-Altman plot illustrating interocular differences in V_30_ rate. Interocular difference is plotted against the average interocular rate for each subject as represented by one circular data point. Long dashed line represent median interocular rate difference; gray small dashed lines represent third and first quartile interocular rate difference; black long dash–dot lines represent upper and lower reference values for 1× annual progression rate; gray double lines represent upper and lower reference values for 2× annual progression rate; black small dotted lines represent upper and lower reference values for 3× annual progression rate.

Interocular differences in progression rates were examined with the Bland-Altman method (Fig. 4 as an example). As shown in Table 2, V_Total_-derived rates had the largest proportion of subjects with interocular differences that were smaller in magnitude than the respective annual progression rate (73.3%). This was followed by V_30_ (64.3%) and OMS (53.8%). In contrast, only 36.4% and 33.3% of interocular differences for V_5_ and BCVA/CS metrics fell within the confines of their annual progression rates.

### Overall Exponential Progression Rates

**Figure 5:**
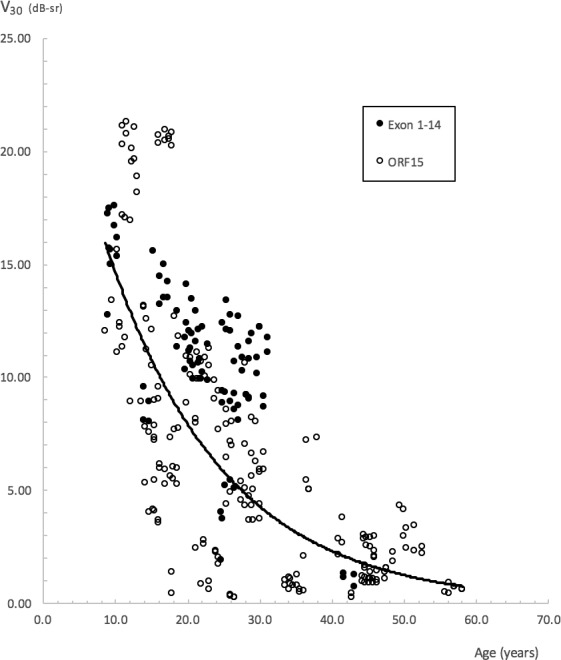
Scatterplot of V_30_ against subjects' age. A common decline for all subjects is represented by the solid exponential line with the equation y = 26.9651 e^−0.0613^^x^. R^2^ = 0.49. The equation is provided only as a guide, as overall progression rates for the study were obtained via the mixed-models method.

Data from all 289 reliable visual fields, together with BCVA and CS data obtained during perimetry visits, were used to calculate overall exponential progression rates for all metrics. Figure 5 shows an example of a scatterplot of V_30_ values plotted against age. Progression rates and half-lives obtained via the mixed-models method are shown in Table 6.

The greatest annual rate of exponential decline was obtained with V_30_ (6.16%), followed by CS (6.12%), V_Total_ (5.78%), BCVA (5.02%), OMS (4.67%), and V_5_ (2.66%). Likewise, the shortest half-lives were obtained with V_30_, CS, and V_Total_ at 10.91, 10.97, and 11.65 years, respectively. A longer half-life of 14.14 years was obtained with BCVA. The longest half-lives were obtained with OMS at 14.50 years and V_5_ at 25.68 years.

A trend toward greater rates of decline in the Exon 1-14 subgroup was seen with OMS and V_Total_, that is, metrics driven by peripheral visual function. Rates of decline for both groups were approximately similar when assessed with V_30_. There was, however, overlap in 95% confidence intervals for rates calculated on the basis of genotype, indicating that genotype-specific differences in rates may not be significant.

### Effects of Age and Genotype on Baseline Visual Function

**Figure 6:**
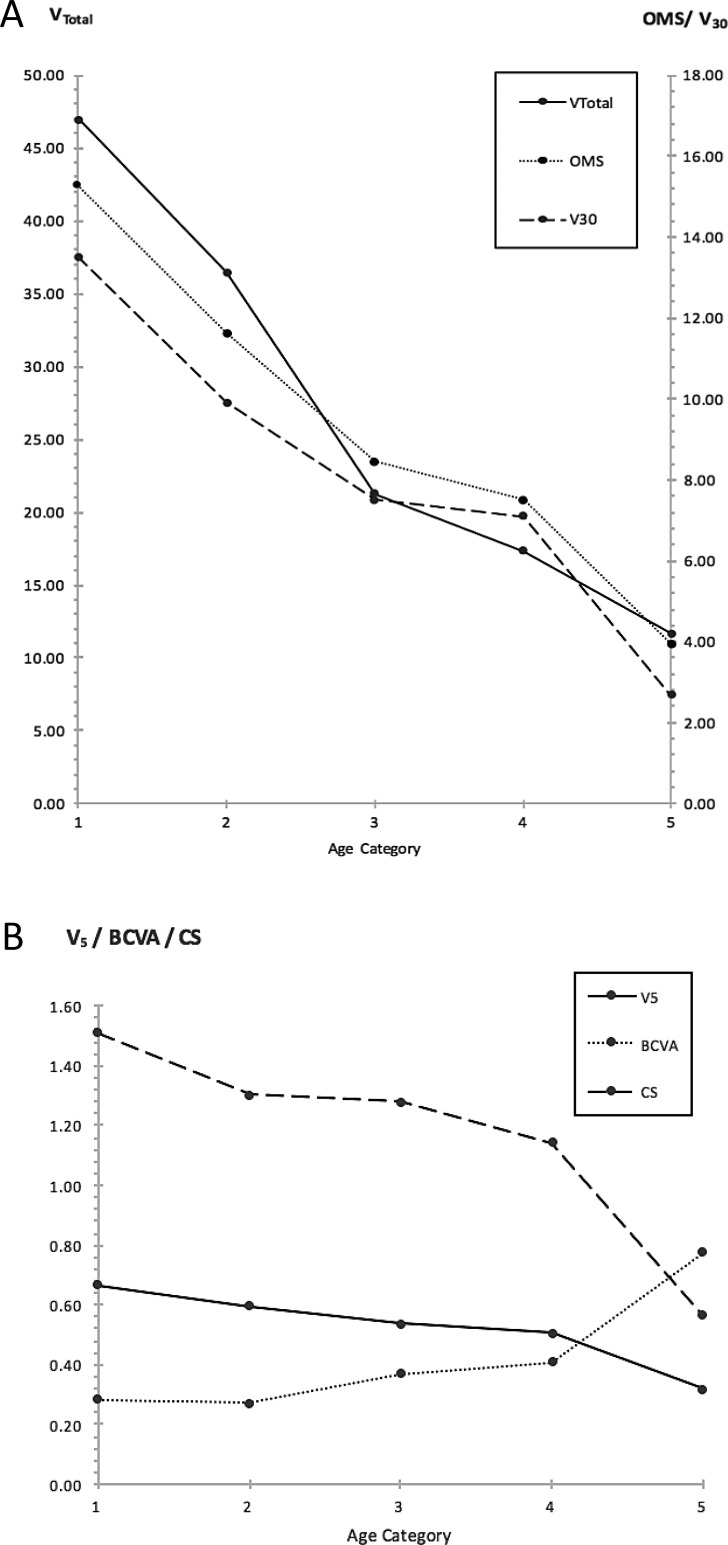
Baseline visual function of subjects as grouped by respective age categories. Visual function characterized by metrics of peripheral function is shown in (A), function characterized by metrics of isolated central function is shown in (B). A decline in peripheral function is evident from the early ages, whereas a decline in central function becomes apparent only in the later-age categories. Age categories: (1) <15 years of age; (2) 15 to 20 years; (3) 20 to 25 years; (4) 25 to <30 years; (5) ≥30 years.

The effects of age and genotype on visual function at baseline were investigated with a 2-way ANOVA, with age and genotype categorized into discrete groups (Table 5). The effects of age were significant on baseline function as characterized by all six metrics (*P* < 0.0001 for CS and perimetry-derived metrics; *P* = 0.0058 for BCVA) with a decline in function evident with increasing age. This decline is clearly shown in Figure 6. For the metrics influenced to varying degrees by “peripheral” retinal function OMS, V_Total_, and V_30_, significant differences in baseline function were most apparent between category 1 and categories 3, 4, and 5, with a clear decline seen within the earlier-age categories. For the metrics of isolated central visual function V_5_, BCVA, and CS, significant differences were seen between category 5 and the younger categories, with decline in function only becoming more apparent during the later-age categories.

Genotype played a significant effect on baseline function as characterized by CS and perimetry-derived metrics, but not on BCVA function. Subjects with mutations in Exon 1-14 had better baseline function. The interaction of both factors on baseline function was not significant.

### Correlations/Associations Between SPID, Baseline Values, Age, and Progression Rates

#### Baseline Values With Age

Associations between baseline metric values and age are shown in Table 4. All metrics correlated strongly and negatively with age at baseline (*r*_s_ ≥ −0.68, *P* < 0.0001 for all, except BCVA with *r*_s_ = 0.62, *P* < 0.0001). The effect of age on baseline function was described above, in detail.

#### BCVA and CS With Perimetry-Derived Metrics at Baseline

The strongest correlations were seen between functional measures of central vision at baseline (Table 4). V_5_ correlated strongly with CS (*r*_s_ = 0.87, *P* < 0.0001) and BCVA (*r*_s_ = −0.78, *P* <0.0001). Correlation between baseline BCVA and CS was also strong (*r*_s_ = −0.80, *P* < 0.0001).

#### Baseline SPID With Interocular Value and Age

Moderate negative correlations reaching statistical significance were seen between the SPIDs of V_Total_, V_30_, V_5_, CS, and their respective baseline values (Table 4). Correlation was strongest for V_5_ (*r*_s_ = −0.60, *P* < 0.0001). Potential associations between baseline SPID and age were investigated as a means of determining whether the degree of interocular symmetry changes with age. With the exception of V_5_ (*r*_s_ = 0.39, *P* = 0.0076), which demonstrated a weak SPID association with age, all others were not statistically significant.

#### Progression Rates With Baseline Values and Age at Baseline

As shown in Table 4, the greatest correlation between progression rates and corresponding baseline function was characterized by V_30_, V_Total_, and OMS (*r*_s_ = 0.64, *P* < 0.0001; *r*_s_ = 0.53, *P* = 0.0007; *r*_s_= 0.49, *P* = 0.0027, respectively). The only significant correlation between progression rate and age at baseline was seen with V_30_ (*r*_s_ = −0.56, *P* = 0.0004). Weaker correlations between progression rates of V_Total_ and OMS with age did not reach statistical significance after Bonferroni correction. All correlations between progression rates with either baseline or age were not significant for metrics representing central visual function.

## Discussion

### Baseline Interocular Symmetry

**Figure 7:**
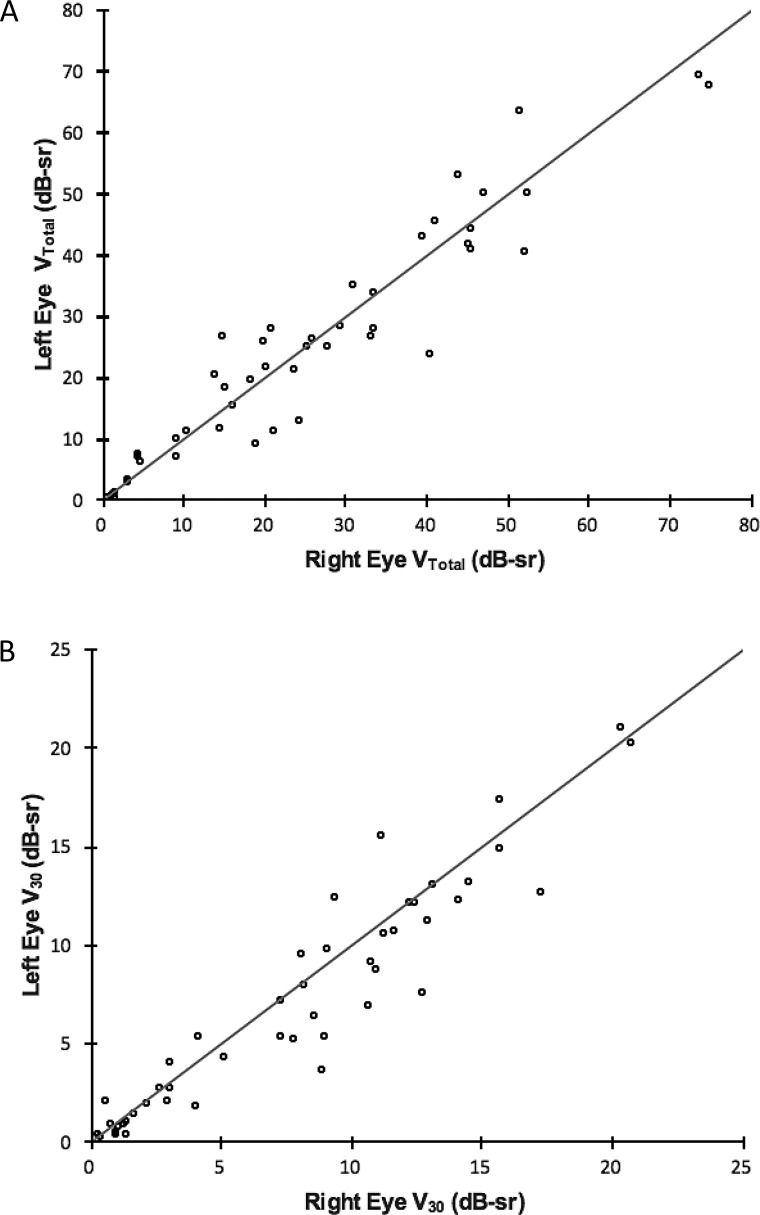
Scatterplots of V_Total_ (A) and V_30_ (B) volumetric metrics at baseline for all study subjects, right eyes corresponding to left eyes. Spearman correlation coefficients—r_s_ = 0.94, P < 0.0001 for V_Total_ and r_s_ = 0.95, P < 0.0001 for V_30_—indicate a very strong and significant interocular correlation for both metrics. Diagonal lines represent the line of equality.

In general, there was overall symmetry at baseline as demonstrated by the small mean interocular differences (Table 1) and further illustrated by the RID values of 1% for V_Total_ and 8% with V_30_. The largest RID of 10% was seen with BCVA. There was, however, significant variation in the level of interocular symmetry or differences across study subjects as illustrated by the wide LOA and the RIV. The RIV was as high as ≥50% with perimetry-derived metrics and >100% with BCVA. Despite this variation, baseline interocular correlations for V_Total_ and V_30_ were very strong with values of 0.94 and 0.95, respectively (Fig. 7). Thus, arguably the use of correlation as a sole method for assessing interocular symmetry is not sufficient to prove the existence of good interocular symmetry in all subjects. As an example, mean V_Total_ in our study was 25 dB-sr with a small mean interocular difference of 0.2 dB-sr. The 95% LOA was, however, as high as 11.5 dB-sr, indicating that an assumption of good interocular symmetry in subjects should not be made without prior inspection of data (Table 1).

Likewise, in a recently published retrospective cross-sectional study of *RPGR* subjects, interocular correlation of visual function with Spearman's correlation is reported as high as 0.98, yet most data points do not lie on the line of equality, indicating that most subjects do not possess similar or identical levels of interocular function.^20^ While originally described for purposes of comparing different methods of measurement, Bland and Altman^42^ give a good description of measurement agreement versus correlation per se, principles of which are equally applicable to analyses of interocular symmetry and have been used by others for this purpose.^48^

As a consequence of finding significant variability in interocular differences between subjects, we quantified the degree of interocular symmetry or differences with the SPID index. In direct comparison to the RIV (a cohort-derived metric), the SPID allows us to quantify each individual subject's interocular difference in relation to his or her baseline function, thereby being more appropriate when comparing visual fields of different magnitudes primarily due to differences in age and genotype.

It is important to distinguish subjects possessing adequate interocular symmetry for purposes of treatment trials, as the untreated “fellow” eye may serve as a control to the treated eye. Furthermore, the use of SPID as an index of interocular symmetry can easily be extended to studies involving other inherited retinal conditions. There is antecedence for untreated “fellow” eyes to be used as controls in treatment trials, for example, in the National Eye Institute Diabetic Retinopathy Study^49,50^ and the Early Treatment Diabetic Retinopathy Study.^51^ It is however important to monitor function and progression of both eyes for a period before treatment, as part of a natural history study, given the concerns about the possibility of eliciting therapeutic effects in the untreated “fellow” eye. In this regard comparisons made with “fellow” eyes in subjects possessing good interocular symmetry will only serve to complement the natural history data obtained in the treated eye albeit before treatment. The provision of contralateral eye data may also be necessary to fulfill potential US Food and Drug Administration requirements for subjects undergoing treatment trials.^52^

### Individual Progression Rates and Interocular Symmetry

Individual progression rates as quantified with BCVA, CS, and V_5_ metrics were small in magnitude. In comparison, more robust rates of progression were obtained with OMS, V_Total_, and V_30_ metrics (V_Total_ > V_30_ > OMS). This is not surprising since the former three metrics provide a measure of central vision that is only affected in the later and advanced stages of disease and hence their use would not be sufficient to sensitively capture disease progression.

As a measure of maximal achievable interocular rate symmetry and to optimize interocular rate symmetry, we determined the proportion of subjects with bilateral trend lines of *R*^2^ ≥ 0.4 with the various metrics. Secondly, we compared the interocular rate differences of these subjects to the corresponding annual progression rate in order to determine the proportion of subjects with interocular rate differences that are smaller in magnitude than the progression rate. With regard to the first measure, the proportion of subjects with bilateral trend lines of *R*^2^ ≥ 0.4 was greatest when perimetry data were analyzed with V_Total_ and V_30_. With regard to the second measure, the highest level of interocular rate symmetry was achieved with V_Total_, followed by V_30_ (Table 2). These two findings indicate that V_30_ and V_Total_ are most suited for use to quantify change in treatment studies if symmetry is regarded as valuable in helping to determine efficacy and safety.

### Cohort Progression

The greatest rates of exponential decline were obtained with V_30_, CS, and V_Total_ metrics, followed by BCVA, OMS, and V_5_ metrics. Pooling all functional data together to allow for exponential analyses allowed us to obtain an arguably truer and precise estimate of progression with the various metrics studied, as subjects spanned a range of ages from 9 to 56 years.

Our exponential BCVA decline rate of 5.02% is greater than the annual decline in visual acuity reported by two studies of *RPGR*-RP subjects (4.0%^17^ and 3.5%^19^). Our perimetry-derived exponential decline rates, in particular OMS rate of 4.7% per annum, is similar to that obtained by Sandberg et al.^17^ who quantified perimetric progression (albeit with kinetic Goldmann V4e perimetry) in a cohort of molecularly confirmed subjects with *RPGR*-associated RP. Sandberg et al.^17^ have found an exponential decline rate of 4.7% per annum, which is greater than the progression previously reported in their *RHO*-RP cohort of 2.9% per annum.^53^ Huang et al.,^18^ in another study of *RPGR*-associated RP subjects, report an annual perimetry (Goldmann V4e) decline rate of 9%; this is the average value from individual rates of only 13 subjects. Bellingrath et al.^20^ have recently published a retrospective cross-sectional study of *RPGR* subjects in which they are unable to adequately assess perimetric progression in patients with increasing disease severity beyond the second decade of life. This may be due to their use of the Goldmann III4e isopter as compared to the more commonly used V4e for kinetic perimetry in RP studies.^12–18,54^

The RF as an arbitrary construct is nevertheless a useful and succinct tool to summarize test reliability with the specific purpose of identifying reliable tests.^55^ The RF threshold for test inclusion (RF ≤ 25) has been used in previous work on RP subjects to good effect.^27^ Additional scrutiny was placed on tests with RF scores between 21 and 25 with the exclusion of tests bearing a false-positive rate exceeding 10% on the basis of the subject being “trigger-happy” (i.e., responsive in the absence of stimuli). The inclusion of unreliable tests can be corrosive to results, as a 10% false-positive answer rate can give rise to an erroneous 1.5-dB increase in mean sensitivity.^56^ The number of tests lost owing to unreliability was however small, as shown in Table 7.

In our study, we additionally relied on the use of *R*^2^ ≥ 0.4 as a method to exclude subjects with great fluctuation in performance, in order to calculate individual progression rates. We included all individual slopes with an *R*^2^ ≥ 0.4 regardless of slope steepness or how quickly or slowly we found their rate of decline to be. By using this approach, we sought to minimize the bias of selecting for subjects with great progression rates—different from the approach taken by others whereby a significant proportion of subjects are excluded on the basis of slow or insufficient progression.^13–15^ In addition, all reliable perimetry data were included into our calculations for overall exponential decline rates. Our approach in determining progression rates of visual function can be applied to other forms of RP, particularly in instances where variability and fluctuation in performance can pose a challenge to the interpretation of progression without bias. However, if we were to reanalyze individual progression rates obtained from the linear trend lines of our subjects with ≥1-year follow-up, disregard our *R*^2^ (goodness-of-fit) criterion, and exclude those with positive rates of change (i.e., improvement in function), we would obtain the following mean percentage annual rates of decline for V_Total_, V_30_, OMS, BCVA, and CS,: 12.3%, 9.4%, 8.1%, 7.6%, and 6.9%, respectively.

### Genotype Correlations

We found a trend toward a greater rate of exponential decline in peripheral function for our Exon 1-14 group when characterized with V_Total_, with an annual rate of 8.07%, almost twice that of the ORF15 rate of 4.91%. Our finding of a possible difference in rates between genotype categories may however have been confounded by our Exon 1-14 subjects being younger than our ORF15 subjects. Mean (SD) age for our Exon 1-14 subjects at time of observation was 22.7 (7.5) years compared to 28.5 (13.4) years for our ORF15 subjects. This, however, warrants further investigation. Interestingly, a similar finding has also been reported by Sandberg et al.^17^ (albeit as a trend that did not reach statistical significance), whereby a greater rate of VF decline has been found in their Exon 1-14 group (4.9%) than in their ORF15 group (4.6%).

### Effects of Age and Genotype on Baseline Values

Age exerted a significant effect on baseline function as characterized by all metrics studied. The decline in baseline function as characterized by the “peripheral” metrics—OMS, V_Total_, and V_30_—is evident early on within the younger-age categories, in comparison to the decline seen with metrics of central function, which only becomes apparent during the later-age categories (Fig. 6). Thus, use of OMS, V_Total_, and V_30_ metrics would be ideal to track functional changes occurring during the earlier stages of disease, in comparison to metrics used to characterize central visual function, which would be better suited to track changes at more advanced stages of disease.

### Correlation Between Metrics of Central Visual Function

BCVA correlated strongly with CS (*r*_s_ = −0.80, *P* < 0.0001). From the perimetry-derived metrics, strongest correlation with BCVA or CS was evident with V_5_ (*r*_s_= −0.78, *P* < 0.0001 and *r*_s_ = 0.87, *P* < 0.0001, respectively). These would be anticipated, given that V_5_ quantifies central visual function as it assesses function within a central 5° radius.

### Correlation Between Progression Rates With Baseline Values and Age

Significant correlations between baseline values and progression rates were found for V_30_, V_Total_, and OMS. Strongest correlation was with V_30_, namely, *r*_s_ = 0.64, *P* < 0.0001. Baseline value and rate correlations for V_Total_ and OMS were *r*_s_ = 0.53, *P* = 0.0007 and *r*_s_ = 0.49, *P* = 0.0027, respectively. The only correlation to reach statistical significance for progression rates and age at baseline was seen with V_30_, namely, *r*_s_ = −0.56, *P* = 0.0004. These findings provide further justification for the consideration of V_30_ and V_Total_ as the two foremost functional metrics in treatment studies, among the six metrics used in our study.

Herein we present the first prospective longitudinal study that comprehensively used various functional metrics to characterize bilateral visual function, progression, and interocular symmetry in subjects with *RPGR*-associated RP (PubMed search on November 19, 2017, with keywords RPGR and perimetry; RPGR and “visual fields”), thereby addressing a current gap in knowledge and providing a much-needed resource to inform patient selection and design of outcome measures in recently commenced *RPGR* treatment trials. We set out to characterize visual function and progression specifically with the use of static perimetry, BCVA, and CS testing. Other modalities can also be used to characterize and complement natural history data and in determining treatment effects with regard to upcoming treatment trials; however, this was not the purpose of our study. Regardless, we do not propose to have made an exhaustive choice of modalities and we accept limitations of our testing strategy after having considered knowledge from previous research together with the practicalities of repeated testing for all the subjects involved in this natural history study. In general, there was good interocular symmetry of visual function; however, we propose the use of SPID as a tool to quantify symmetry given the significant level of variation that exists across individuals. We showed that progression in the earlier stages of the condition is best assessed by metrics that characterize peripheral function as compared to metrics of central function. We believe our findings will contribute to current levels of care by enabling clinicians to provide disease-specific prognostic information to affected individuals. Finally, we anticipate that the methods of assessing individual progression rates and symmetry used in this study can be applied to studies involving other forms of RP and inherited retinal conditions.
